# An Attenuated Coxsackievirus B3 Vector: A Potential Tool for Viral Tracking Study and Gene Delivery

**DOI:** 10.1371/journal.pone.0083753

**Published:** 2013-12-30

**Authors:** Jun Zeng, Xiao xuan Chen, Jian ping Dai, Xiang feng Zhao, Gang Xin, Yun Su, Ge fei Wang, Rui Li, Yin xia Yan, Jing hua Su, Yu xue Deng, Kang sheng Li

**Affiliations:** 1 Department of Microbiology and Immunology, Shantou University Medical College, Shantou, Guangdong, People’s Republic of China; 2 Department of Endocrinology, The First Hospital of Yichang, Three Gorges University College of Medicine, Yichang, Hubei, People’s Republic of China; University of Kansas Medical Center, United States of America

## Abstract

Cardiomyocytes are quite resistant to gene transfer using standard techniques. We developed an expression vector carrying an attenuated but infectious and replicative coxsackievirus B3 (CVB3) genome, and unique *Cla*I-*Stu*I cloning sites for an exogenous gene, whose product can be released from the nascent viral polyprotein by 2A^pro^ cleavage. This vector was tested as an expression vehicle for green fluorescent protein (GFP). The vector transiently expressed GFP in cell cultures for at least ten passages and delivered functional GFP to the infected cardiomyocytes for at least 6 days. Moreover, the recombinant viruses showed virulence attenuation *in vitro* and *in vivo*. The findings suggest that the recombinant CVB3 vector could be a useful tool for viral tracking study and delivering exogenous proteins to cardiomyocytes.

## Introduction

Gene therapy is a promising treatment of choice for myocardial diseases [1]–[3]; however, cardiomyocytes are quite resistant to gene transfer by standard techniques [4], and therefore developing an efficient gene delivery method remains a major challenge for cardiac gene therapy.

Due to high expression efficiency and ability to transfect a wide range of cell types, recombinant viral vectors are commonly used for gene transfer. Clinical application of viral vectors is however limited by potential unwanted effects, for example with retroviral vectors, there is a risk of vector genome integration into the host chromosome, inducing tumorigenic mutations. Coxsackievirus B3 (CVB3) has a significant advantage over other viral vectors by replicate in nondividing cells, such as myocytes. Although lentiviruses can also replicate in nondividing cells and thus are considered as potential viral vectors for cardiac gene therapy [5], these viruses may cause concern because they are derived from human immunodeficiency virus type 1 (HIV1). Coxsackievirus-adenovirus receptor (CAR) and decay accelerating factor (CD_55_) have been shown to enable coxsackievirus attach and enter the cell [6]. CAR is expressed at low levels in normal adult heart but it is increased during cardiomyopathy or in a damaged heart [7]. Therefore, CVB3 could be exploited as a vector to deliver exogenous genes to the heart or as a viral tracking vector.

In this study, we developed an attenuated CVB3-based vector that can deliver exogenous genes to cardiomyocytes and express in situ, with reduced virulence in mice.

## Results

### Characteristics of Recombinant Plasmids and Viruses

**Figure 1:**
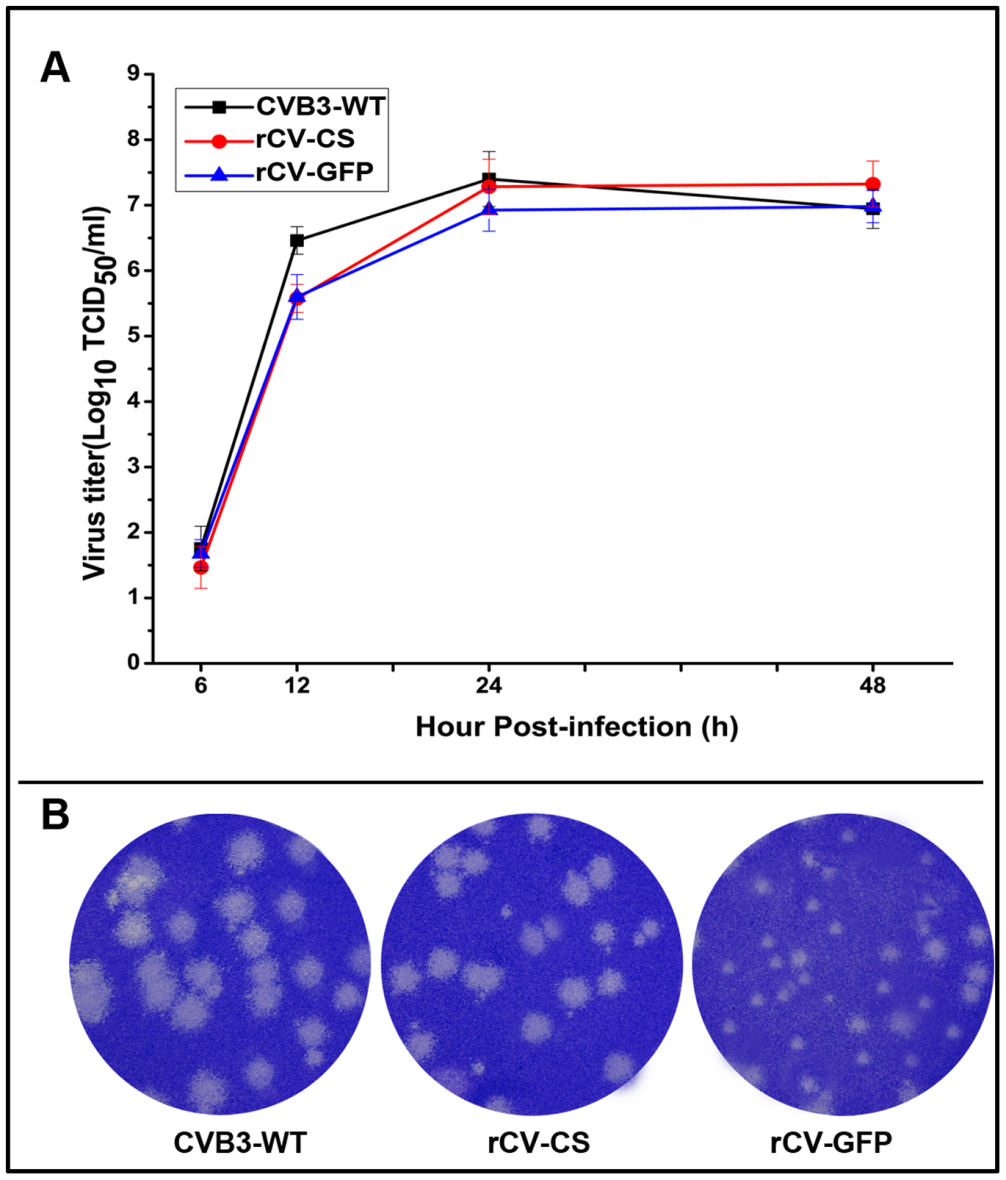
In vitro characteristics of recombinant viruses. (A) Viral growth kinetics in Vero cells infected with viruses at an MOI of 0.01 analyzed by TCID_50_ assay. Data from three independent experiments are shown. (B) Plaque morphology of viruses on Vero cells.

pCV-CS had unique cloning sites (*Cla*I and *Stu*I). pCV-GFP was created on pCV-CS backbone. The infectious recombinant virus (rCV-CS) or recombinant virus expressing GFP (rCV-GFP) was isolated from the supernatants of transfected COS-7 cells. In Vero cells, both recombinant viruses showed growth kinetics equivalent to that of CVB3-WT (Figure 1A). Exogenous insertion did not compromise the vector viability. However, recombinant viruses exhibited different plaque morphologies: while CVB3-WT’s plaques were heterogeneous in sizes (2–11 mm in diameters), rCV-CS and rCV-GFP only produced smaller plaques with a diameter of 2–3 mm (Figure 1B), showing attenuated virulence.

### Functional Stability of GFP *in* *vitro* and *in vivo*

**Figure 2:**
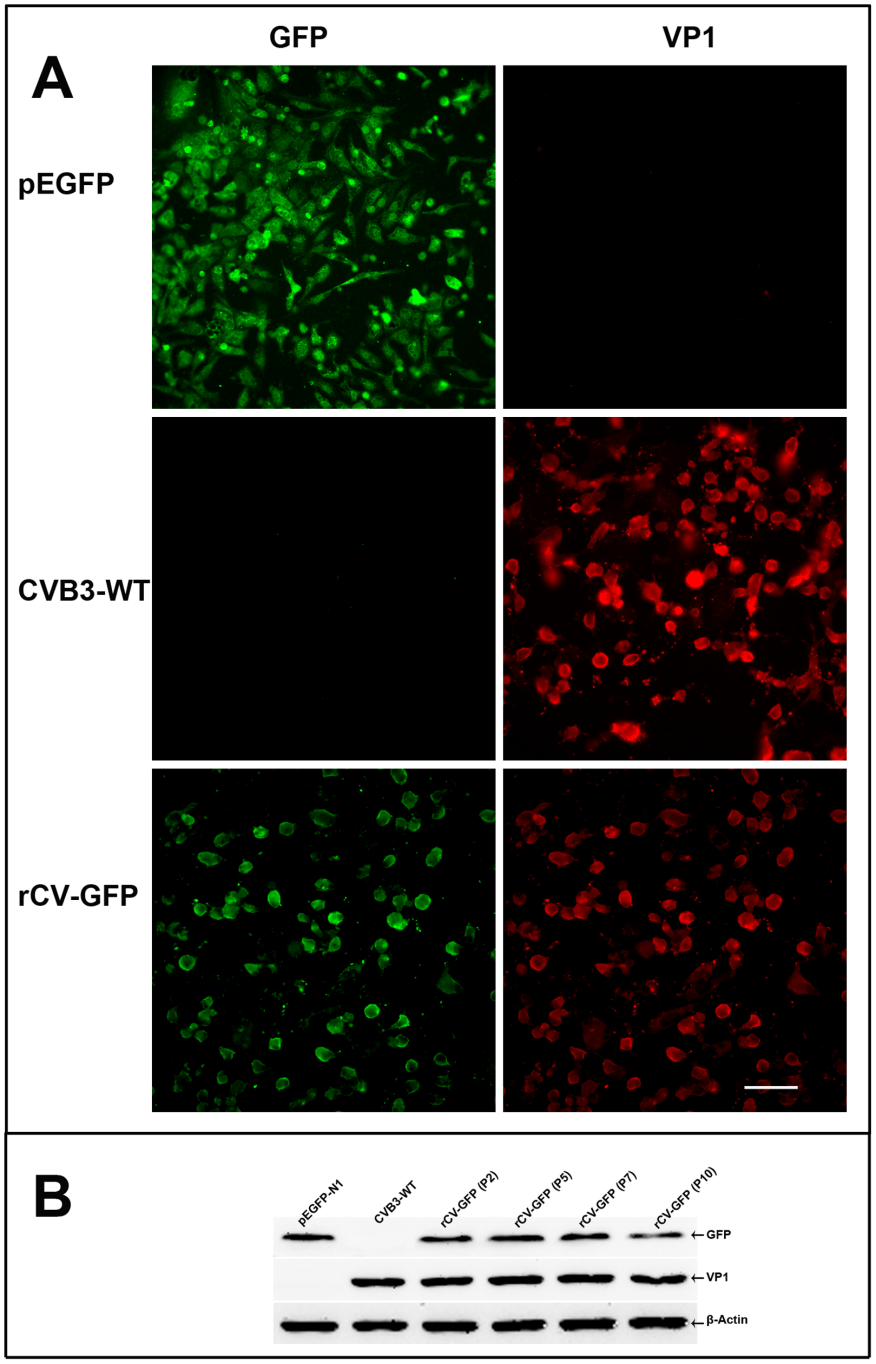
Expression of GFP and VP1 in cultured cells. (A) Vero cells infected with rCV-GFP or transfected with pEGFP-N1 (control), showing green fluorescence (GFP) and red fluorescence (viral protein, probed with anti-enteroviral VP1 antibody). (B) GFP expression from rCV-GFP examined at passages 2 to 10 by immunoblotting using anti-GFP antibody, anti-enteroviral VP1 antibody, or anti-*β*-actin antibody, with pEGFP-N1 and CVB3 as controls. Scale bar 50 µm.

**Figure 3:**
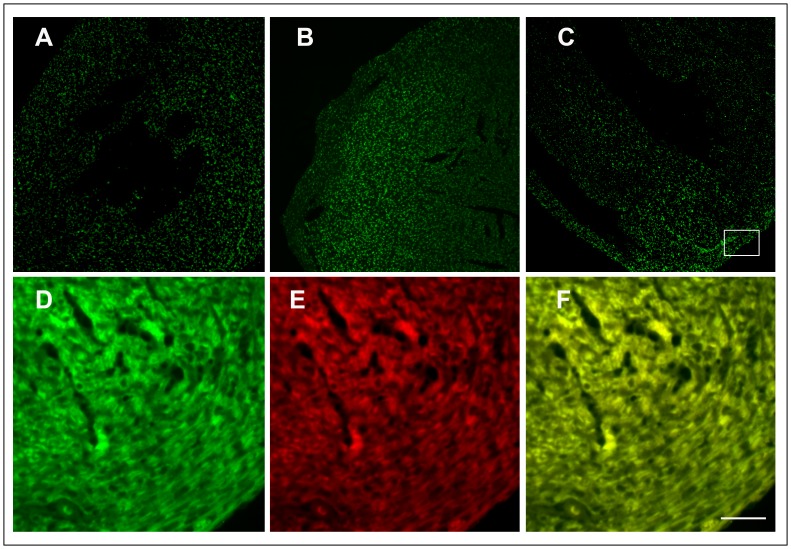
GFP expression from rCV-GFP in infected mice heart. Expression of GFP (green fluorescence) in heart at day 2 (A), day 4 (B), and day 6 (C) post-infection. Higher magnification of inset showing GFP (D), viral capsid protein in red fluorescence (E), and their co-localization (F). Scale bar, 100 µm.

Functional GFP was detectable in both infected and transfected Vero cells (Figure 2A). Since viruses could change during prolonged tissue culture or during the course of infection in mice, *in-vitro* stability of rCV-GFP in the culture was examined at the 2, 5, 7, and 10 passages by Western Blot analysis of GFP expression. The GFP released from the viral polyprotein was detectable up to the tenth passages (Figure 2B). The *in-vivo* genetic stability was examined in mice infected with 10^5^ TCID_50_ of rCV-GFP via an intraperitoneal route. From days 2 to 6 post-infection, we found GFP co-localizing with VP1 in the heart and pancreas as visualized by immunofluorescence (Figure 3), demonstrating the infectivity and stability of rCV-GFP and GFP.

### Attenuated Virulence of Recombinant Viruses in Mice

Sequence of rCV cDNA (cloned from the passage 5), in comparison with CVB3 WT genome, revealed that there were 23 nucleotide changes resulting in 10 amino acid replacements after serial passage (Table 1).

**Table 1:** Comparison between wild type and attenuated CVB3 genomes.

Viral genome	Position	CVB3-WT → CV	
		Nucleotide changes	Amino acid changes
**5′ NTR**	125	G→A	
	127	C→T	
	529	C→T	
	578	G→A	
	586	C→T	
**VP2**	1445	A→G	Lys→Glu
**VP3**	1861	G→A	
	2024	A→T	Ser→Cys
	2276	G→A	Ala→Thr
	2430	C→T	Thr→Ile
	2438	G→C	Glu→Gln
	2448	T→A	Phe→Tyr
	2467	C→T	
**VP1**	2685	A→C	Glu→Ala
	2690	G→A	Glu→Arg
	2691	A→G	
**2A**	3324	C→T	Ala→Val
**2B**	4006	G→A	
**2C**	4996	T→C	
**3C**	5412	C→T	Thr→Met
**3D**	7163	T→C	
	7273	A→G	
**3′ NTR**	7334	C→T	

There were 23 nucleotide changes resulting in 10 amino acid replacements in attenuated CV genome.

**Figure 4:**
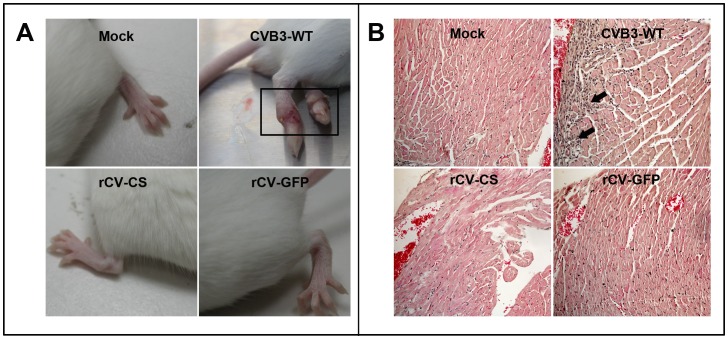
Virulence attenuation of recombinant viruses in mice. Mice were inoculated with 10^5^ TCID_50_ of viruses and sacrificed on scheduled days. (A) Mice in CVB3-WT group showing poor general condition and skin ulceration from days 8 to 14 post infection. (B) Hematoxylin and eosin histology of hearts infected with CVB3-WT, rCV-CS, or rCV-GFP, showing myocardial inflammation in CVB3-WT infected heart (arrows).

Viral ability to invade tissues, cause pathology (inflammation), and replicate in host were investigated clinically and histologically. All the mice infected with rCV-CS, rCV-GFP, or CVB3-WT at 10^5^TCID_50_ via an intraperitoneal route survived until day 14 (the end point of experimentation). CVB3-WT-infected mice showed poor general condition, skin ulceration (Figure 4A), and significant body weight loss, whereas mice infected with recombinant viruses appeared more active and gained body weight (Figure S1). The general condition was consistent with histological findings as myocarditis was observed exclusively in CVB3-WT-infected mice, not in rCV-CS or rCV-GFP-infected mice (Figure 4B).

**Figure 5:**
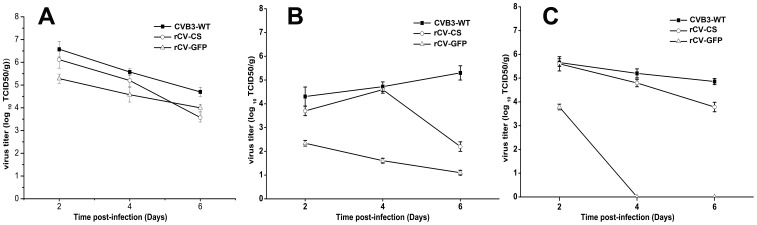
Titers of viruses in tissues of mice. The titers of each virus in the pancreas (A), heart (B), and liver (C) were determined by TCID_50_ assay on days 2, 4, and 6 post-infection.

As shown in Figure 5, the wild type as well as recombinant viruses were able to infect heart, liver, and pancreas, with most efficient replicability in pancreas, followed by live and heart. Attenuated replication of recombinant viruses was observed mainly in heart especially with rCV-GFP.

## Discussion

We have developed an efficient method to express an exogenous protein in cardiomyocytes *in vivo* using an attenuated CVB3 as a gene delivery vector. During recent years, some members of the picornavirus group have been studied in view of their ability to express foreign proteins or peptides. Of them, recombinant CVB3 vectors have shown potential as a viral vector for vaccination or gene delivery.

CVB3 belongs to the *picornavirus* family, genus *enterovirus*. CVB3 is noneveloped, containing a single-stranded positive-sense RNA genome of approximately 7400 nt with one open reading frame [8], [9]. After translation, the viral polyprotein is cleaved by viral proteases into four capsid proteins and seven functional proteins such as RNA polymerase and proteases (2A^pro^ and 3C^pro^) [10]. Previously, foreign coding sequences have been inserted into CVB3 genome at two sites. One is immediately downstream to the translation start site, from which the exogenous protein can be released by 3C^pro^ cleavage. This site allows foreign insert but suffers from instability of the insert with reduced viral replication [11]. The other insertion site is at the junction between the viral VP1 and 2A genes, where the foreign protein can be released from the rest of viral proteins during translation with the help of an artificial proteolytic cleavage site recognized by 2A^pro^. This strategy permits the isolation of a replication-competent recombinant virus carrying foreign genes of various sizes [12], [13].

**Figure 6:**
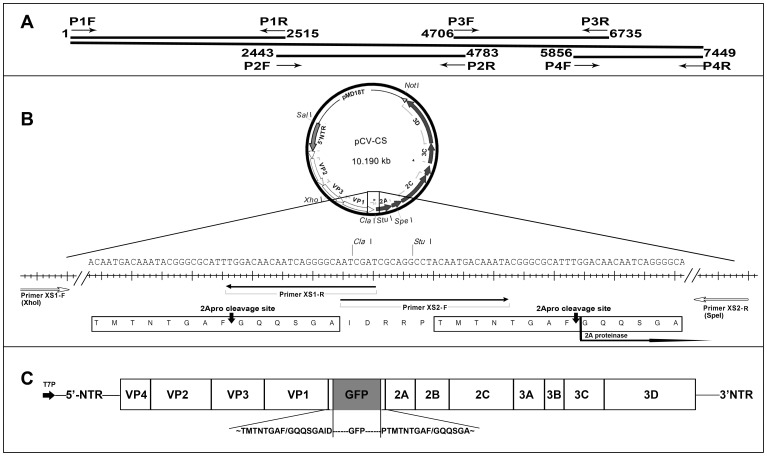
Schematics of recombinant CVB3 expression vector. (A) Primers used to generate sub-genomic CVB3 fragments (see Table 2) and the assembly strategy of the complete genome of CVB3. (B) Elaborate map of a duplicated 2Aproteinase cleavage sites and two unique cloning sites (*Cla*I and *Stu*I) in pCV. (C) Relative position of an exogenous gene GFP in the viral genome of pCV-GFP. During viral RNA translation and polyprotein proteolytic processing, GFP will be excised from the mature protein at 2Aproteinase cleavage sites. Abbreviations: T7P, T7 RNA polymerase-dependent promoter; NTR, Non-translated region of the CVB3 genome; VP, Viral capsid protein.

We employed the latter strategy with some modifications. Our vector carries a full-length cDNA copy of CVB3 genome with unique *Cla*I-*Stu*I cloning sites and two 2A^pro^ cleavage sites between VP1 and 2A: one before and the other after the *Cla*I-*Stu*I site (Figure 6). Therefore, the expressed protein from an exogenous gene at the *Cla*I-*Stu*I site (GFP in this study) can be released from the viral polyprotein by 2A^pro^ cleavage, allowing viral capsid formation to proceed. Due to its convenience, stability, and less harmless nature, GFP was used in this study to examine the ability of the vector to express exogenous genes, and to track the functional stability of the expressed proteins *in vitro* and *in vivo.*

Meyer et al. [14] have made a GFP expression recombinant CVB3 in which the capsid coding P1-region (VP4 to VP1) was replaced with a GFP gene. This vector allowed the viral RNA replication and expression, however, such virus was defective and did not produce infectious progeny viruses *in vivo*. In two other studies, CVB3 was designed to carry exogenous genes of up to 320 nt from the site between VP1 and 2A [12] [13]. By comparison, our study is the first report of GFP expression from the site between VP1 and 2A but the GFP-coding sequence (720 nt) was much larger, and it was maintained and expressed in the CV genome for at least ten passages in vitro (Figure 2) and functional GFP was detectable in the infected tissues in mice (Figure 3).

Attenuated CVB3 strains from serial passages in cells or mouse tissues have reportedly not caused inflammation in previous studies [15]. Attenuation of CVB3 was also achieved in this study by serial passage in Vero cells. As expected from the altered plaque morphology, we observed attenuated virulence of recombinant viruses (rCV-CS and rCV-GFP) in mice, which was exhibited clinically by better general conditions, no skin ulceration, and body weight gain, or histologically by less inflammation in the hearts at day 6 post-infection, compared to the wild type virus (Figure 4). We found no inflammation in mice liver and pancreas either infected with CVB3-WT or recombinant viruses (data not shown). Some nucleotide changes in the 5′-NTR have been shown to reduce viral translation efficiency and some amino acid changes in viral structure proteins (VP4 to VP1) and supposedly interrupt the viral binding step [15].

Major advantage of the viral vector system presented here is its attenuated virulence in mice. rCV derived from CVB3-WT, acquired multiple mutations in the 5′-NTR and viral coding regions after serial passage (Table 1). The 5′-NTR of picornaviruses play an important role in not only translation, but also replication, and contributes to viral pathogenesis and tissue tropism. A previous report indicated that mutations in internal ribosomal entry site (IRES, 432–639 nt of 5′-NTR) affect the viral proteins replication [16]. Our sequence analyses shown 3 site mutations in the IRES of recombinants compared with WT-CVB3, this result may provide a likely explanation for viral growth kinetics of recombinants virus are lower than that of CVB3-WT. However, we cannot exclude other possible mechanism, such as mutations in VP1 and VP3 may affect viral receptor attachment function. In this study, we found 5 amino acids change in recombinant viruses compared with CVB3-WT. Other investigators have recently suggested that a single amino acid changes in VP3 permit significant shifts in viral receptor and gain tropism for new cell types [17]. Although we do not know the specific mutations responsible for the observed attenuated phenotype, at least the smaller plaque morphology of rCV could be due to deficient attachment of progeny viruses to uninfected cells despite near-wild type replicability as shown in the one-step viral growth curve. More studies are required to clarify this issue.

Because of the relatively mild outcome from the wild type CVB3 infection, we did not determine LD_50_, instead, we examined virus titers in different organs such as heart, pancreas and liver. Although the precise mechanisms for virus organ tropism are not known, it is clear that CVB3 does infect other tissues, such as pancreas and liver as shown in previous reports [18], [19] That viral titers in the pancreas and liver were even higher than the titer in the heart in this study (Figure 5) may be accounted for by the intraperitoneal infection route, and is consistent with the findings that viral titer of the wild type as well as recombinant viruses appeared self-limiting in all organs examined, except in the heart where self-enhanced replication of the wild type virus was observed until day 6 post infection. Carrying an exogenous gene in rCV-GFP appears to further restrain replication especially in the heart and liver.

In summary, this study presents a recombinant coxsackievirus B3 for use as a novel gene transfer vector that can carry and transiently express exogenous proteins. rCV-GFP, in particular, could be a useful tool for coxsackievirus tracking study and this vector could be further exploited for gene therapy purpose.

## Materials and Methods

### Cell Lines and Virus

COS-7 cells and Vero cells were purchased from the Cell Bank of the Chinese Academy of Sciences (Shanghai, China) and were maintained in DMEM (Gibco, USA) supplemented with 10% fetal bovine serum (HyClone, USA), 100 U/ml penicillin and 100 µg/ml streptomycin at 37°C in a CO_2_ incubator. The CVB3 (Nancy strain) was kindly provided by Dr. Qihan Li (Institute Of Medical Biology, Chinese Academy Of Medical Science and Peking Union Medical College). Viral stocks were prepared from Vero cells when the cytopathic effect (CPE) reached 95%. The viral supernatant from three freeze-thaw cycles was stored in aliquots at −80°C.

### CVB3 Attenuation

To generate attenuated viral strains, CVB3 wild type (Nancy, CVB3-WT) was serially passaged, plaque cloned, and amplified in Vero cells. Viral strain with smaller plaque morphology at the passage 5 was used for recombinant viral constructs.

### Construction of Recombinant Infectious Viral Vectors, pCV, pCV-CS, and pCV-GFP

Viral RNA was prepared from the viral cultural supernatant using NucleoSpin RNA Virus (Macherey-Nagel, Germany) and used in first-strand synthesis with SuperScript II (Invitrogen), following the manufacturers’ instructions.

Complete genomic sequence of attenuated CVB3 was generated from four overlapping CVB3 genomic fragments that were separately amplified from the attenuated CVB3 strain by PCR using Phusion High-Fidelity DNA Polymerase (Finnzymes, Thermo scientific, USA) and four pairs of primers - P1F/R, P2F/R, P3F/R, and P4F/R (Table 2). PCR profiles were as follows: denaturation at 98°C for 30 sec, followed by 30 cycles of 98°C for 30 sec and annealing at 63°C for 30 sec (for fragments 1 and 2) or 58°C for 30 sec (for fragment 3), or no annealing step for fragment 4, extension at 72°C for 2 min, and the final extension at 72°C for 5 min. PCR products separated on 1.2% agarose gels were purified using an E.Z.N.A. Gel Extraction Kit (Omega, Bio-Tek).

**Table 2:** PCR Primers used in this study.

Primers	Sequences (5′→3′)	Use
P1F	CC*GTCGAC* **TAATACGACTCACTATAGGG**TTAAAACAGCCTG(*Sal*I)	pCV
P1R	TCCCTATAGCGGCTGTTA	
P2F	GACACTCCTTTCATTTCGC	
P2R	CCAAGGCTCTGCTATCTGACACG	
P3F	TCACCTCACCGTTTGTCTTG	
P3R	AATGTAGTTTGTCTCTTTGTGCG	
P4F	GGTACTGGGTATCCATGTTG	
P4R	TA*GCGGCCGC*(T)_15_CCGCACC(*Not*I)	
XS1-F	GTA*CTCGAG*TGTTTTTAGTCGGACG*(Xho*I*)*	pCV-CS
XS1-R	CG*ATCGAT*TGCCCCTGATTGTTGTCCA(*Cla*I)	
XS2-F	CA*ATCGAT*CGC*AGGCCT*ACAATGACAAATA(*Cla*I and *Stu*I)	
XS2-R	CC*ACTAGT*GATTCTTTCAGGAGG(*Spe*I)	
GFP-F	TC*ATCGAT*ATGGTGAGCAAGGG(*Cla*I)	pCV-GFP
GFP-R	TA*AGGCCT*CTTGTACAGCTCGT(*Stu*I)	

The rectangle showing the T7 promoter sequence.

Overlap extension PCR was performed with 100 ng each of four purified genomic fragments, High-Fidelity DNA Polymerase, and dNTPs for 10 cycles of denaturation at 98°C for 30 sec and annealing at 63°C for 30 sec, extension at 72°C for 2 min followed by amplification with primers P1F and P4R for 30 cycles of 98°C for 30 sec, 63°C for 30 sec, and 5 min at 72°C. The final PCR products (∼7.45 Kbp) were gel purified and cloned into a plasmid pMD18, generating pCV (Figure 6A). The sequence of the attenuated CVB3 genome (CV) was verified at BGI (Beijing Genomics Institute, China), and is shown in comparison with the CVB3 WT sequence in Table 1.

pCV was engineered to carry two unique cloning sites (*Cla*I and *Stu*I) after VP1, flanked by one artificial 2A protease (2A^pro^) cleavage site and, in pCV between the last encoded viral capsid protein VP1 and 2A^pro^. The construct was obtained by overlap PCR amplification from two overlapping fragments (XS1 and XS2) of pCV: with primers XS1-F and XS1-R to generate fragment XS1 (2008–3322 nt) with *Xho*I site upstream, and with primers XS2-F and XS2-R to generate fragment XS2 (3281–3845 nt) with a *Spe*I site downstream. Overlap extension PCR was performed with 100 ng each of purified XS1 and XS2 fragments, High-Fidelity DNA Polymerase, and dNTPs for 10 cycles of denaturation at 98°C for 30 sec and extension at 72°C for 2 min, followed by amplification with primers XS1-F and XS2-R for 30 cycles of 98°C for 30 sec, 57°C for 30 sec, and 2 min at 72°C. The final PCR product was digested with *Xho*I and *Spe*I and was ligated into *Xho*I-*Spe*I restricted pCV to generate pCV-CS (Figure 6B). The orientation of insert was verified by sequencing.

A GFP-expressing CV vector was constructed by cloning a GFP gene (GenBank accession no. U55762.1), which was amplified from pEGFP-N1 (TaKaRa, China) with primers GFP-F and GFP-R (Table 2). The GFP gene was digested and ligated into *Cla*I*-Stu*I site of pCV-CS, generating pCV-GFP (Figure 6C). Two flanking 2A^pro^ sites allow the release of GFP from the nascent viral polyprotein.

### Preparation and Characterization of Recombinant Viruses

pCV, pCV-CS, and pCV-GFP were transfected into COS-7 cells using FuGene 6 (Roche, Mannheim, Germany). Three days after transfection, replication-competent recombinant viruses (rCV, rCV-CS, and rCV-GFP) were harvested. Viral growth curve, titration, and plaque assay were performed essentially as described previously [10]. Plaques were visualized after fixing the cultures with 10% formalin for 30 min and staining with 1% crystal violet.

### GFP Expression in Vero Cells

Vero cells were infected with rCV-GFP or CVB3-WT at an MOI of 0.1 or transfected with pEGFP as control. At 24 h post-infection or transfection, cells were fixed and viral capsid protein VP1 was detected by fluorescence assay using primary antibody, mouse anti-VP1 (Dako, Germany), and secondary antibody, Cy3 conjugated goat anti-mouse IgG (Invitrogen, USA). GFP fluorescence was observed under a fluorescence microscope using the FITC filter.

### Western Blot Analysis of Viral Proteins and GFP in Infected Cells

Vero cells were inoculated with different passages of rCV-GFP or CVB3-WT at an MOI of 1 or were transfected with pEGFP-N1 and incubated for 24 h, washed with PBS, and lysed in Laemmli buffer on ice. The lysates were separated in 12% SDS-polyacrylamide gels and electroblotted onto PVDF membrane (Roche). Blots were blocked for 2 h in TBS-T (Tris-Buffered Saline, 0.05%Tween-20) containing 5% dry milk, washed with TBS-T, and incubated for 2 h with 1∶5000 dilution of the primary mouse anti-VP1 antibody. Blots were washed as described above and incubated with the Horseradish peroxidase-conjugated rabbit anti-mouse IgG (CST, USA) at a dilution of 1∶10000. The same procedure was used to detect GFP expression by using mouse anti-GFP (Beyotime, China) as primary antibody. Detection was performed using an ECL Kit (Amersham, GE Healthcare) in Fluor Chem FC2 System (Alpha Innotech, USA).

### Animal Experiments

Three-week-old male Balb/c mice (from Guangdong Medical Laboratory Animal Center, Foshan, China) were fed a standard diet and sterile water in a pathogen-free room and distributed randomly into 4 groups with 12 mice in each group: 1) uninfected control, 2) CVB3-WT, 3) rCV-CS, and 4) rCV-GFP. Mice were inoculated intraperitoneally with 200 µl sterile 100 mM NaCl containing 10^5^ TCID_50_ of virus or without virus as control. Mice (n = 3, per day in each group) were euthanized at days 2, 4, and 6 post-infection. Tissues were fixed in 10% formalin for histology or frozen in liquid nitrogen for determination of virus titer. All animal experimental protocols were approved by Shantou University Medical College and were in accordance with the guide for the care and use of laboratory animals (National Academy Press, 1996).

### Histology

Paraffin sections (5 µm) were stained with haematoxylin-eosin or deparaffinized and dehydrated through a graded alcohol series for immunostaining. Sections were immunolabeled with anti-viral VP1antibody as described earlier. The stained sections were observed under a Nikon 90i microscope (Nikon, Japan) and the images were captured with a SPOT CCD microscope digital camera (Diagnostic Instruments Inc., Sterling Heights, MI).

### Viral Titer in Mouse Tissues

Heart, pancreas, and liver tissues were weighed, homogenized in complete cell-culture medium, and used TCID_50_ assay performed on Vero cells. Virus titer was expressed as TCID_50_ g^−1^.

## Supporting Information

Figure S1 Body weight change. Body weights were taken every other day, beginning at day 0, the day of with or without virus inoculation until the end of the study.
(TIF)
