# Supplementary figures and images for: An Attenuated Coxsackievirus B3 Vector: A Potential Tool for Viral Tracking Study and Gene Delivery
Source: PLoS One. 2013 Dec 30;8(12):e83753. doi: 10.1371/journal.pone.0083753 (PMC3875476; doi:10.1371/journal.pone.0083753)

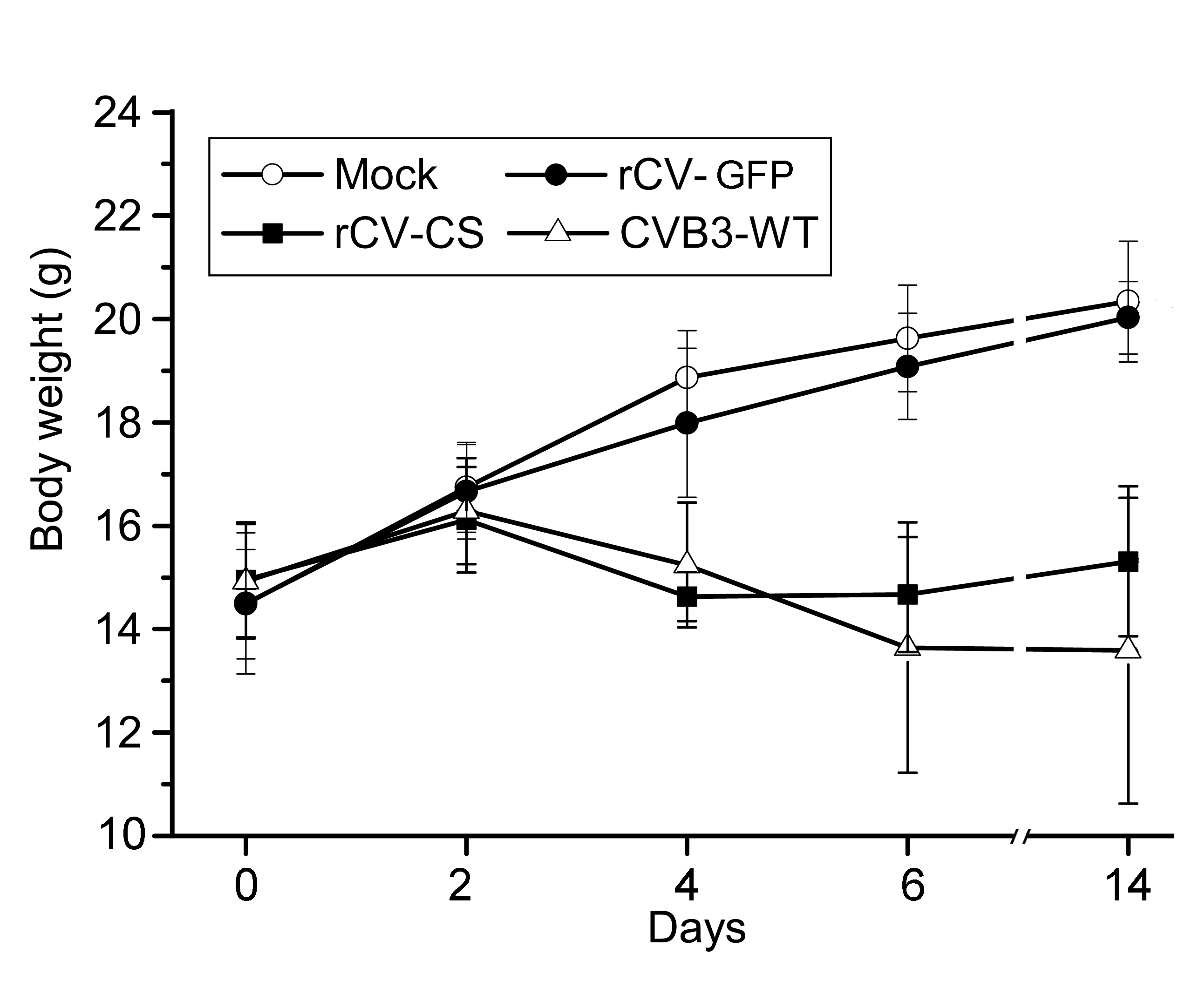

Supplement: Figure S1 — Body weight change. Body weights were taken every other day, beginning at day 0, the day of with or without virus inoculation until the end of the study. (TIF) [file pone.0083753.s001.tif]
